# The efficacy and safety of opioid-free anesthesia combined with ultrasound-guided intermediate cervical plexus block vs. opioid-based anesthesia in thyroid surgery—a randomized controlled trial

**DOI:** 10.1007/s00540-023-03254-9

**Published:** 2023-09-23

**Authors:** Zhi Liu, Congjie Bi, Xingguo Li, Ruonan Song

**Affiliations:** 1 Department of Anesthesiology, Dalian Municipal Central Hospital, Dalian, Liaoning China; 2 China Medical University, Shenyang, China

**Keywords:** Opioid-free anesthesia, Thyroid surgery, Intermediate cervical plexus block, Nausea and vomiting, s-Ketamine

## Abstract

**Purpose:**

In the context of the current comfort medicine and enhanced recovery after surgery, there is a demand for a new anesthesia method to reduce adverse reactions and accelerate recovery after surgery. This randomized controlled trial aimed to compare the efficacy and safety between opioid-free anesthesia (OFA) combined with ultrasound-guided intermediate cervical plexus block (ICPB) and opioid-based anesthesia in patients after thyroid surgery.

**Methods:**

In this study, 75 patients scheduled for thyroid surgery under general anesthesia were randomly allocated into two groups. The primary outcome included the incidence of nausea within 24 h after surgery. The main secondary outcomes included the incidence of vomiting and the visual analog score (VAS) scores within 24 h after surgery as well as the quality of recovery 40 questionnaires (QoR-40) scores 24 h after surgery.

**Results:**

In the OFA group, the incidence of postoperative nausea was 6.1%, compared to 39.4% in the control group (*p* = 0.001). No patient presented with postoperative vomiting in the OFA group, while 15.2% of patients suffered from postoperative vomiting in the control group (*p* = 0.063). The VAS scores of patients in the postanesthetic care unit (PACU) and 2 h, 4 h, and 6 h after surgery were lower in the OFA group, and the difference is statistically significant. Besides, the VAS scores of patients at rest (*p* = 1.000) and during swallowing (*p* = 1.000) 24 h after surgery were comparable.

**Conclusion:**

Compared with opioid-based anesthesia, the OFA combined with the ultrasound-guided ICPB can better improve patients' postoperative recovery, reduce nausea, and decrease pain scores.

**Trial registration:**

Chinese Clinical Trial Regisrty, ChiCTR2200056344, https://www.chictr.org.cn

## Introduction

In the past 20 years, significant advancements have been made in the application of opioids to pain control. However, with the widespread use of opioids, there is a growing awareness of their adverse effects. Among them, opioid-induced constipation and nausea are the most common side effects [1]. In addition, opioids can also lead to gastrointestinal motility disorders, physical dependence, and tolerance in patients, as well as hyperalgesia [2, 3]. Furthermore, the use of opioids may delay extubation and cause muscle fatigue, hypoventilation, ileus, and urinary retention [4].

Opioid-free anesthesia (OFA) is a multimodal anesthesia management strategy combining various non-opioid agents and/or technologies. Lidocaine exerts an analgesic effect by silencing ectopic discharges, suppressing inflammatory processes, and regulating inhibitory and excitatory neurotransmission. Also, it is effective in improving rehabilitation, shortening hospital stays, promoting early bowel movements, reducing postoperative nausea and vomiting (PONV), and reducing nociceptive and/or cardiovascular responses to surgical stresses [5, 6]. As an α_2_ adrenoceptor agonist, dexmedetomidine exerts sedative and analgesic effects through the locus coeruleus center and the posterior horn of the spinal cord, and it has sympathetic neurolysis [7]. S-Ketamine, as an S-isomer of ketamine, has a double potency compared with ketamine, and it may induce fewer side effects [8]. Therefore, OFA is undoubtedly a more attractive option for high-risk patients with PONV.

For thyroid surgery, PONV due to opioids and pain at the incision site are the main postoperative complications associated with anesthesia that may inflict sufferings on patients [9]. After thyroidectomy, the incidence of PONV can reach 70–80% in the absence of preventive antiemetic treatment [10]. As a common complication of thyroid surgery, postoperative pain, especially within 24 h after surgery, can prolong the hospital stay and may even result in re-hospitalization [11, 12].

Ultrasound-guided intermediate cervical plexus block (ICPB) is a regional nerve block technology that can provide adequate analgesia in head and neck surgery. Compared with the deep cervical plexus block, ICPB has a lower incidence of postoperative serious complications [13]. Compared with the superficial cervical plexus block, ICPB provides a better 24-h analgesia effect after surgery under the same postoperative complications [14]. At the current stage, OFA has not been applied to open surgery with large wounds, such as thyroidectomy. Therefore, we conducted a randomized controlled trial in thyroid surgery to identify whether OFA combined with ultrasound-guided ICPB could meet the surgical needs and reduce the incidence of PONV and pain scores.

## Methods

### Study and ethics

Ethical approval for the study was granted by the Ethics Committee of Dalian Municipal Central Hospital (yn-2021-085-01). It has been registered with the Chinese Clinical Trial Regist (ChiCTR2200056344). The patient was included from February 2022 to September 2022. The written informed consent was obtained from all participants.

This study was designed as an assessor-blinded parallel-group randomized controlled trial. It was conducted to evaluate whether opioid-free general anesthesia combined with ultrasound-guided ICPB can meet the needs of thyroid surgery. Besides, this combined strategy was compared with traditional opioid-based general anesthesia in terms of their effects on PONV and pain after thyroid surgery.

### Inclusion and exclusion criteria

The inclusion criteria of the trial included ASA I-II patients aged 18–70 years who underwent elective thyroid surgery. The exclusion criteria included patients with a history of neck surgery; patients with a history of infection at the surgical site; patients with coagulation dysfunction; patients with giant thyroid tumors in the neck; patients with chronic pain who need to be treated with opioids after surgery; patients who were chronically treated with beta-blockers with pulse rate < 50 beats/min; patients with preoperative oxygen saturation measured by pulse oximetry (SpO_2_) less than 95%; patients who were allergic or contraindicated to this test; patients who did not agree to sign the subject statement.

### Randomization and blinding

The patients were allocated randomly to the OFA group or the control group according to a computer-generated random number table. Allocation to the treatment group was performed using the sealed opaque envelope technique. Sealed envelopes were marked as the OFA group or the control group and were opened only when the patient entered the operating room. The patient, outcome assessor, and nurses in the postanesthetic care unit (PACU) and surgical ward were blinded to the grouping. Due to the significant differences in anesthesia methods, anesthesiologists knew the allocation of patients. Before surgery, patients were instructed to complete pain scoring on the VAS rating scale. 0 points represented no pain, and 10 points represented the most severe pain. All operations were performed with a standardized technique. An investigator who was blind to the grouping was trained to interview patients and fill in the quality of recovery 40 questionnaires (QoR-40) [15] form 24 h after surgery.

### Outcomes

The primary outcome included the incidence of nausea within 24 h after surgery. We use the simplified PONV impact scale by Myles et al. to record PONV [16]. This validated interview consists of two questions.** (**Q1. Have you vomited or had dry- retching?; Q2. Have you experienced a feeling of nausea? If yes, has your feeling of nausea interfered with activities of daily living, such as being able to get out of bed, being able to move about freely in bed, being able to walk normally, or eating and drinking?) When the score for question one is 1, we believe that the patient has experienced postoperative vomiting, and when the score for question two is 1, we believe that the patient has experienced nausea.

The secondary outcomes included the incidence of intraoperative hemodynamic adverse events, defined as MAP exceeding ± 20%, pulse rate > 100 beats/min or < 40 beats/min; QoR-40 scores 24 h after surgery; pain scores assessed by VAS in the PACU and 2 h, 4 h, 6 h, and 24 h after surgery; utilization rate and dosage of remedial analgesics after extubation; postoperative hypoxemia, defined as a SpO_2_ < 95%; urinary retention, defined as no urination 24 h after surgery; postoperative ileus, defined as the absence of flatus or stools 24 h after surgery; complications of ICPB; dizziness, headache, and pruritus.

### Anesthesia process

The anesthetic dosage in the OFA group was determined based on previously reported methods and its feasibility was assessed in a pilot series before this study [17–22].

After the patient entered the operating room and the venous access was opened, general anesthesia was induced using etomidate 0.3 mg/kg and remifentanil 1-2 μg/kg (pumping completed within 60 s) in the control group, while patients in the OFA group were premedicated with dexmedetomidine 1 μg/kg over 10 min, and then induced with etomidate 0.3 mg/kg, s-ketamine 0.5 mg/kg, lidocaine 1.5 mg/kg and maintained with dexmedetomidine 0.5 μg/kg/h, s-ketamine 0.25 mg/kg/h, and propofol 3-4 mg/kg/h. Patients in the control group were maintained with propofol 4-6 mg/kg/h and remifentanil 0.05–0.2ug/kg/min. Rocuronium bromide 0.6 mg/kg was used in both groups for muscle relaxation. Endotracheal intubation was performed in both groups after pre-oxygenation. For better blinding, all patients' necks were treated with a dressing after intubation.

**Fig. 1:**
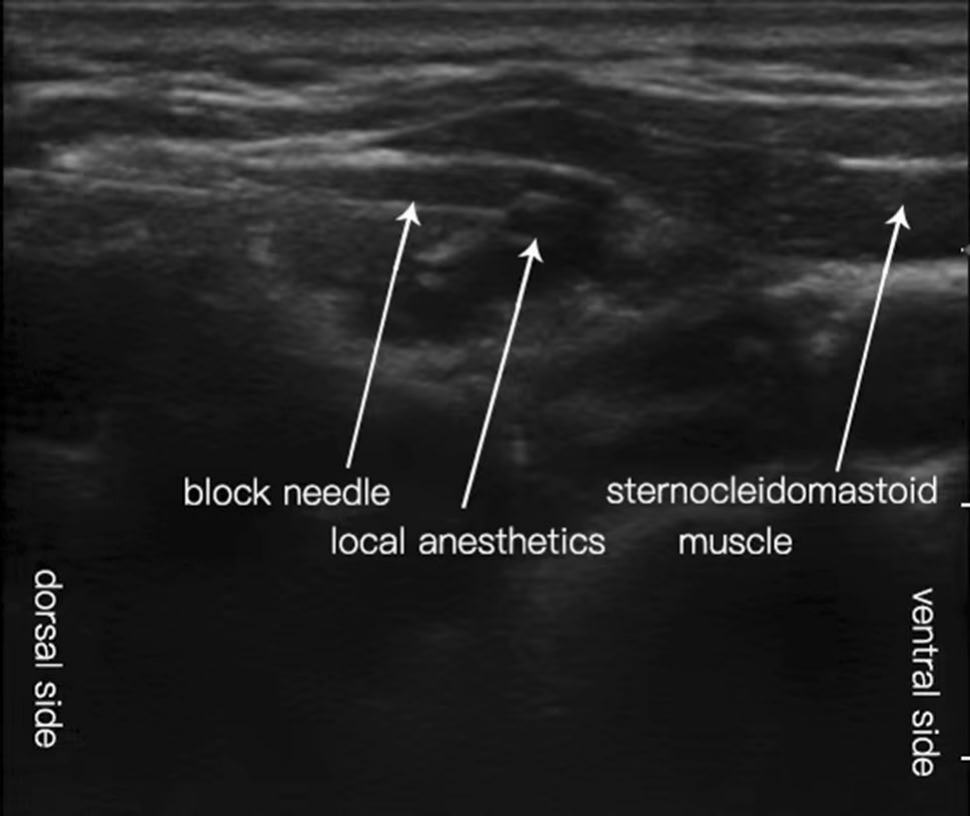
Intermediate cervical plexus block

After intubation, patients in the OFA group received ultrasound-guided ICPB performed by an experienced anesthesiologist. The ICPB procedures described below were similar to the method described in a previous study [22]. Specifically, a sensor probe was transversely placed on the midpoint of the sternocleidomastoid muscle, so that the tapered posterior edge can be located in the middle of the screen (at the level of the cricoid cartilage, where the external jugular vein passed through the sternocleidomastoid process). Then based on the in-plane technology, a blocking needle was pushed into the deep plane of the sternocleidomastoid muscle, near the superficial cervical plexus, under the investment fascia (sternocleidomastoid-trapezius fascia), and immediately above the scalene groove. After negative aspiration, 7.5 mL of 0.25% bupivacaine with 5 µg/mL of dexmedetomidine was deposited under the fascia after feeling the click or ‘pop’ on piercing this fascia. Subsequently, the same method was applied to local anesthesia on the other side (Fig. 1). We added dexmedetomidine to the bupivacaine solution to reduce the possible cardiotoxicity caused by bupivacaine [23] and prolong the sensory block time [24]. Possible complications of ICPB were recorded after surgery. Intraoperative monitoring indicators included ECG, pulse rate, pulse oximetry, non-invasive blood pressure, EtCO_2_, and Narcotrend D2-E1. The withdrawal time was stopped according to the pharmacokinetic characteristics of the drug.

Patients in both groups were ventilated with air/oxygen mixed inhalation (FiO_2_ 35%, tidal volume 7–10 ml/kg, and respiratory rate 10–14 min^−1^) to maintain EtCO_2_ between 35 and 45 mmHg and SpO_2_ between 95 and 100%. Patients in the control group received dezocine 0.15 mg/kg to reduce postoperative pain. In addition, all patients received a dual intravenous antiemetic strategy of 10 mg azasetron and 5 mg dexamethasone during surgery.

Intraoperative hemodynamic parameters were recorded every 5 min. Baseline values were taken 5 min after induction and a 20% rise from baseline in the mean arterial pressure (MAP) or pulse rate (PR) ≥ 100 beats/min prompted the administration of a 40 μg remifentanil bolus in the control group vs. 1 mg nicardipine or 1 mg metoprolol in the OFA group. The selection of blood pressure reduction methods for the two groups was based on the results of the pre-experiment. During the operation, if hypertension occurred, we first thought that it was caused by insufficient analgesia. Therefore, in the control group, remifentanil was given first to control blood pressure, and good hypotensive effect was achieved in the pre-experiment. However, in the pre-experiment of the OFA group, when hypertension occurred during surgery, increasing the dosage of anesthesia or administering non-opioid analgesics did not achieve satisfactory analgesic effects. On the contrary, when we administered antihypertensive drugs, we achieved better antihypertensive effects, so we believed that intraoperative hypertension in the OFA group was caused by side effects of s-ketamine. In case of uncontrollable hypertension, sevoflurane should be inhaled to maintain hemodynamic stability during surgery. Patients with uncontrollable hypertension were excluded and the total number of these patients was recorded. Uncontrollable hypertension was defined as that when remifentanil was pumped or hypotensive drugs were injected intravenously and the efficacy disappears, the blood pressure rose to more than 20% of the basic value for three consecutive times. When the MAP decreased by over 20%, patients in both groups were provided with norepinephrine (8 ug). Besides, 0.5 mg of atropine was given when the pulse rate was less than 40 times/min. The number of patients with adverse hemodynamic events in the two groups was recorded. Additionally, regular doses of neostigmine and atropine were adopted to block the residual neuromuscular block. Extubation was performed when the patient reached a normal spontaneous breathing mode. The surgical duration was defined as the time from the first incision to the completion of suture. The anesthesia duration was defined as the time from the beginning of anesthesia induction to the withdrawal of endotracheal intubation. The extubation duration was defined as the time between the end of surgery and endotracheal extubation. After extubation, the patient was transferred to the PACU.

In the PACU and ward, all patients received oxygen inhalation only when SpO_2_ was lower than 95%, and they were provided with 2.5 mg of dezocine if the VAS scores ranged from 4 to 6 (moderate pain) in the resting state vs. 5 mg if the VAS scores were greater. In addition, these patients were provided with 1 mg of droperidol in case of nausea. These patients were transferred to the ward from the PACU after at least a 30-min stay and when the Aldrete score was ≥ 9 [25].

### Statistical analysis

The sample size was calculated according to the incidence of nausea within 24 h after surgery in 20 patients before this experiment (the OFA group: 5%; the control group: 40%). The power analysis results of PASS software (90% power and a 5% significant level) suggested that each group should incorporate 31 patients. Considering the twenty percent dropouts, a total of 75 patients were included in the two groups in this study.

Categorical variables were reported as number of cases (proportion) and analyzed using the Chi-square test or Fisher's exact test. Continuous variables were expressed as mean ± standard deviation or median (interquartile range). Normally distributed variables were analyzed using the Student's *t* test and non-normally distributed variables were analyzed using the Mann–Whitney *U* test. Bonferroni method was used to correct the *p* value after multiple comparisons. All statistical analyses were conducted using SPSS statistics 26.0. The two-sided *p* value < 0.05 was considered statistically significant.

## Results

### Patient characteristics

**Fig. 2:**
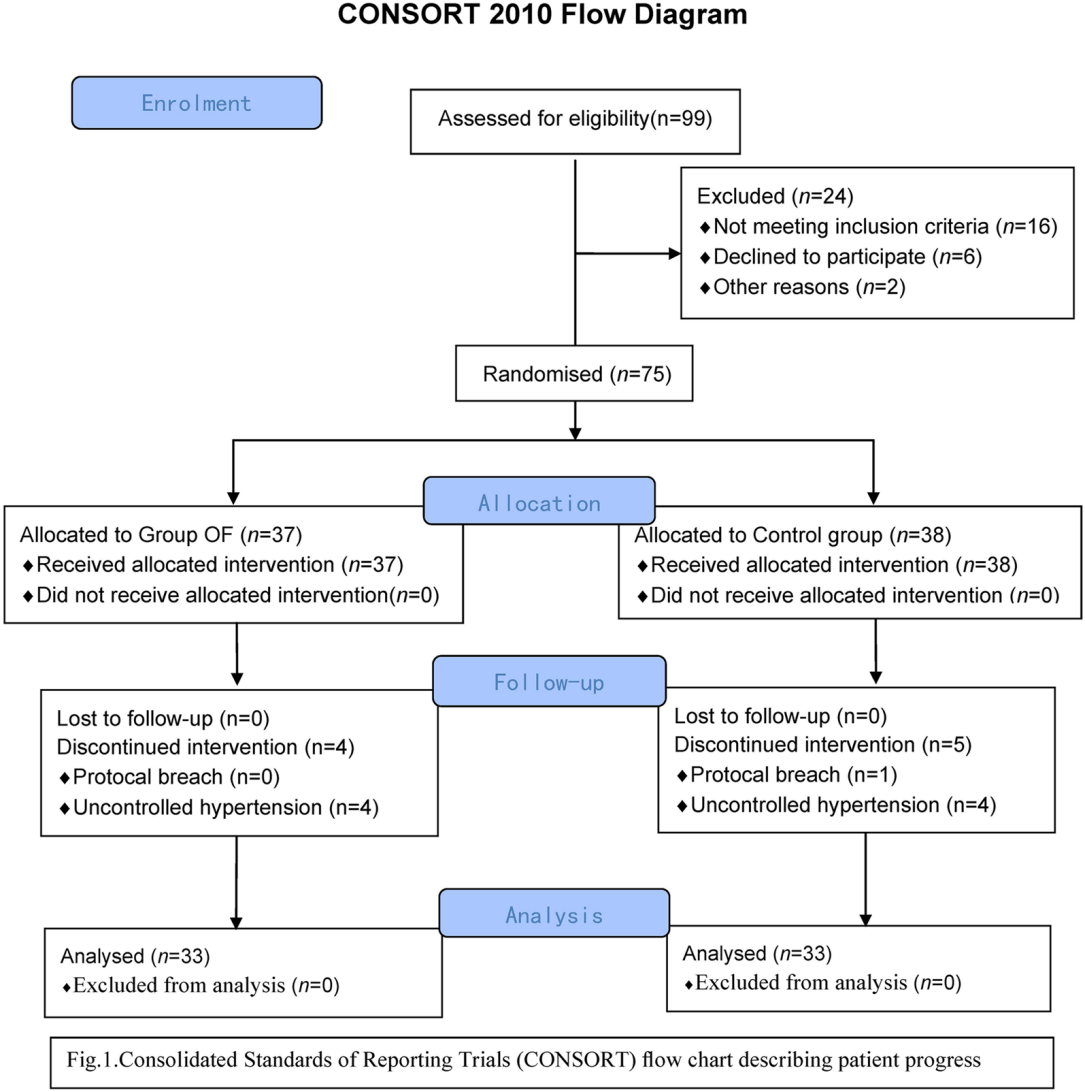
CONSORT 2010 flow diagram

In this study, we enrolled 75 patients undergoing elective thyroid surgery with general anesthesia in Dalian Central Hospital. Among them, 1 patient who took antiemetic drugs in advance was excluded from the control group due to fear of PONV. In addition, there were 4 patients, respectively, in the OFA group and the control group receiving sevoflurane due to uncontrollable hypertension during surgery, and hence they were excluded. Thus, the analysis results were obtained based on the 66 remaining patients (Fig. 2). The clinical characteristics of patients in the two groups are presented in the table (Table 1).

**Table 1:** Characteristics of the patients at baseline

	OFA group (*n* = 33)	Control group (*n* = 33)	*p *value
Gender (M/F)	7/26	4/29	0.322
Age	48.8 ± 9.9	49.9 ± 12.1	0.707
ASA (I/II)	17/16	15/18	0.622
BMI	24.5 ± 2.3	24.7 ± 2.5	0.675
History of smoking: *n* (%)	2 (6.1)	2 (6.1)	1.000
History of previous PONV: *n* (%)	3 (9.1)	1 (3.0)	0.606
History of motion sickness: *n* (%)	8 (24.2)	11 (33.3)	0.415
Surgery time, min	83.0 (63.5–96.0)	92.0 (73.0–108.0)	0.256
Anesthesia time, min	114.0 (98.5–137.0)	113.0 (86.5–128.5)	0.812
MAP, mmHg	92.0 (85.0–103.5)	90.0 (87.5–100.5)	0.404
PR, beats/min	70.0 (64.0–78.0)	70.0 (65.0–78.0)	0.724
Total dose of remifentanil, mg	1.14 (0.94–1.48)	–	–
Surgical procedure			
Partial thyroidectomy	12 (36.4)	15 (45.5)	0.453
Radical thyroidectomy	20 (60.6)	14 (42.4)	0.139
Total thyroidectomy	1 (3.0)	4 (12.1)	0.352

ASA: American Society of Anesthesiologists; PONV: postoperative nausea and vomiting; BMI: body mass index; MAP: mean arterial pressure; PR: pulse rate

^a^Continuous variables are presented as means ± SD or median (25th percentile–75th percentile)

^b^Categorical variables are presented as frequency (%) or numerical value/ numerical value

### Study endpoints

#### Postoperative nausea and vomiting

Postoperative nausea occurred in 2 patients (6.1%) in the OFA group (*p* = 0.001) and 13 patients (39.4%) in the control group. Postoperative vomiting was not observed in the OFA group (*p* = 0.063) but occurred in 5 patients (15.2%) in the control group. However, there was no significant difference in this statistical result (Table 2).

**Table 2:** Postoperative nausea and vomiting

	OFA group (*n* = 33)	Control group (*n* = 33)	*p* value
Nausea at PACU, *n* (%)	2 (6.1)	4 (12.1)	1.000^b^
Nausea at 2 h postoperatively, *n* (%)	0	7 (21.2)	0.082^b^
Nausea at 4 h postoperatively, *n* (%)	0	7 (21.2)	0.082^b^
Nausea at 6 h postoperatively, *n* (%)	0	10 (30.3)	0.003^b^
Nausea at 24 h postoperatively, *n* (%)	0	1 (3.0)	1.000^b^
Nausea at any time, *n* (%)	2 (6.1)	13 (39.4)	0.001
Vomiting at PACU, *n* (%)	0	0	–
Vomiting at 2 h postoperatively, *n* (%)	0	2 (6.1)	1.000^b^
Vomiting at 4 h postoperatively, *n* (%)	0	2 (6.1)	1.000^b^
Vomiting at 6 h postoperatively, *n* (%)	0	4 (12.1)	0.609^b^
Vomiting at 24 h postoperatively, *n* (%)	0	1 (3.0)	1.000^b^
Vomiting at any time, *n* (%)	0	5 (15.2)	0.063

*OFA* opioid-free anesthesia, *PACU* postanesthesia care unit

^a^Categorical variables are presented as frequency (%)

^*b*^*p *-value corrected by Bonferroni method

#### VAS scores, analgesic requirements, and QoR-40 scores

**Fig. 3:**
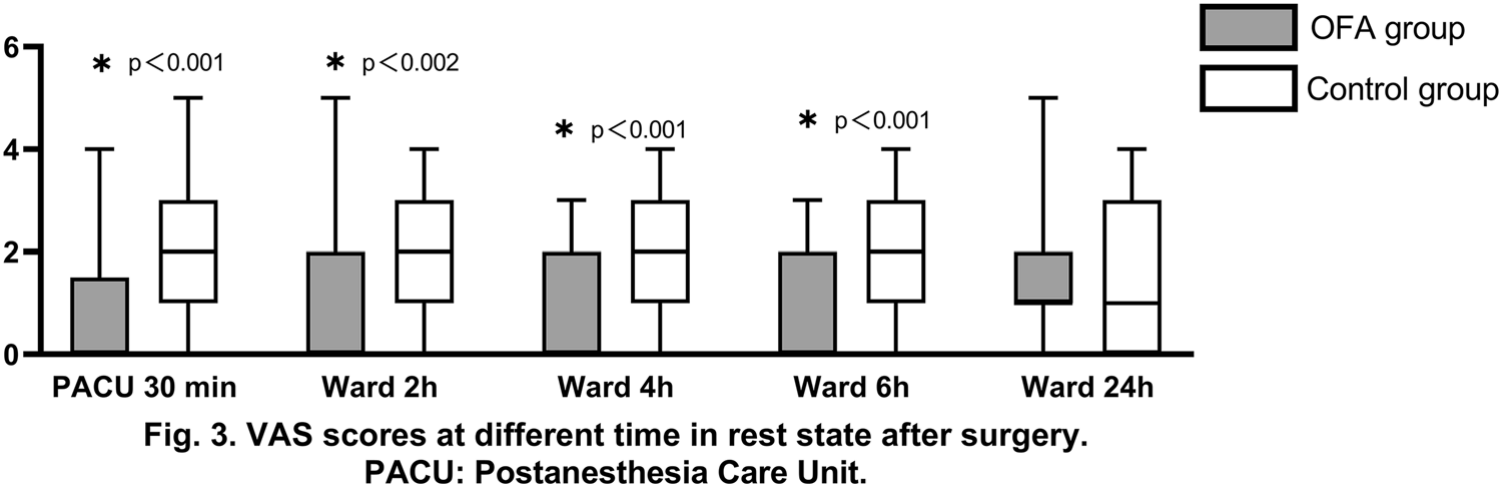
VAS scores at different time in rest state after surgery

**Fig. 4:**
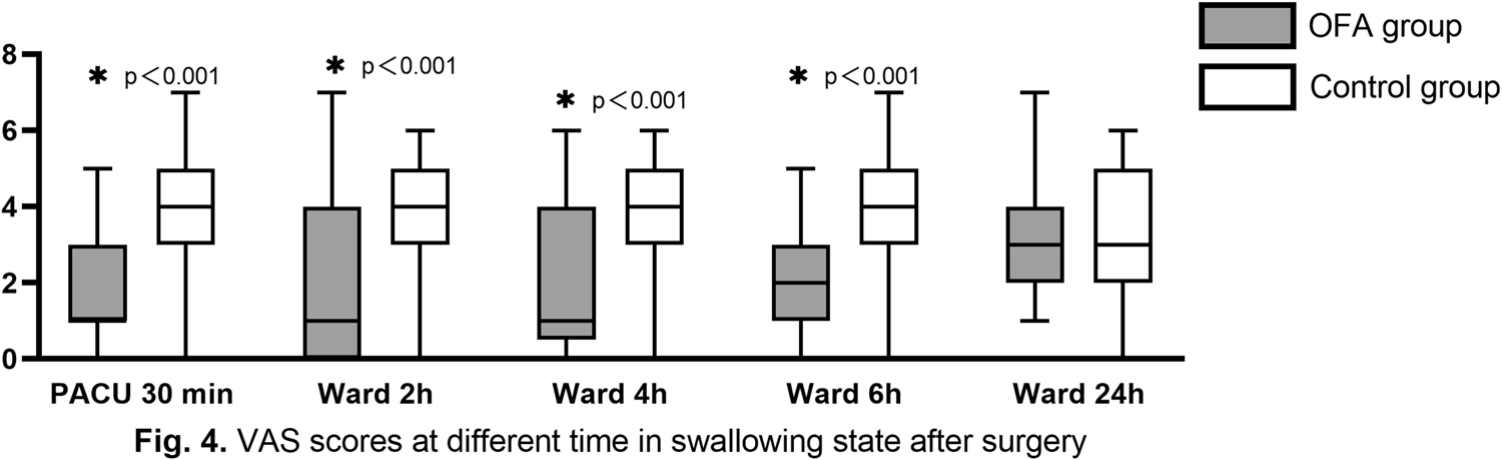
VAS scores at different time in swallowing state after surgery

There was a statistically significant difference in the VAS score of patients between the resting and swallowing states observed in the PACU and 2 h, 4 h, and 6 h after surgery. During swallowing, the median VAS score in the OFA group was 1 in the PACU and 2 h and 4 h after surgery; this value was 2 6 h after surgery. In contrast, the median VAS score in the control group was 4 in the PACU and 2 h, 4 h, and 6 h after surgery. However, the VAS score in the OFA group and the control group at rest and during swallowing 24 h after surgery was (1.0 [1.0–2.0] vs. 1.0 [0–3.0]; *p* = 1.000) and (3.0 [2.0–4.0] vs. 3.0 [2.0–5.0]; *p* = 1.000), respectively. There was no significant difference between the two groups **(**Figs. 3, 4**).**

In the PACU, there was 1 (3.0%) patient in the OFA group and 7 (21.2%) patients in the control group who received postoperative remedial analgesics (*p* = 0.059). In the ward, there was 1 (3.0%) patient in the OFA group and 8 (24.2%) patients in the control group who received postoperative remedial analgesics (*p* = 0.031) (Table 3).

**Table 3:** Remedial analgesics, QoR-40 and incidence of intraoperative hemodynamic adverse events

	OFA group (*n* = 33)	Control group (*n* = 33)	*p *value
Patients receive dezocine in PACU or ward, *n* (%)	2 (6.1)	10 (30.3)	0.011
Total postoperative dezocine consumption (mg) [PACU or ward]	0 (0–0)	0 (0–2.5)	0.013
QoR-40 scores	188.0 (184.0–192.0)	181.0 (174.0–187.0)	0.001
Intraoperative adverse events			
MAP > 20%, *n* (%)	11 (33.3)	7 (21.2)	0.269
MAP < 20%, *n* (%)	5 (15.2)	13 (39.4)	0.027
PR > 100 beats/min, *n* (%)	5 (15.2)	4 (12.1)	1.000
PR < 40 beats/min, *n* (%)	0	0	–
Norepinephrine, ug	0 (0–0)	0 (0–8.0)	0.037

*PACU*: postanesthesia care unit, *MAP*: mean arterial pressure, *PR*: pulse rate

^a^Continuous variables are presented as means ± SD or median (25th percentile–75th percentile)

^b^Categorical variables are presented as frequency (%)

The QoR-40 score 24 h after surgery was significantly higher in the OFA group than in the control group (188.0 [184.0–192.0] vs. 181.0 [174.0–187.0]; *p* < 0.001) (Table 3).

#### Intraoperative and postoperative adverse events

The incidence of adverse events in intraoperative hemodynamics is shown in Table 3. The incidence of intraoperative hypotension in the OFA group (*n* = 5, 15.2%) was significantly lower than that in the control group (*n* = 13, 39.4%; *p* = 0.027). The median number of noradrenaline administration was (*n* = 0, 0–0) in the OFA group and (*n* = 0, 0–8.0) in the control group (*p* = 0.037). There was no statistically significant difference in the incidence of hypertension and tachycardia between the two groups. In addition, there was no patient with PR < 40 times/min. There was no statistically significant difference in the extubation duration, the first flatus duration, the incidence of urinary retention, and the incidence of ileus between the two groups (Table 4). In the OFA group, 3 patients had transient postoperative hypoxemia in the PACU, which was improved after oxygen inhalation treatment. No patient had hypoxemia in the ward. Moreover, no other adverse effects related to ICPB were observed in patients of both groups.

**Table 4:** Incidence of postoperative adverse events

	OFA group (*n* = 33)	Control group (*n* = 33)	*p *value
Time for extubation, min	5.0 (3.0–11.0)	5.0 (3–8.0)	0.347
Time for first flatus, h	11.2 ± 5.0	12.0 ± 6.7	0.640
Urinary retention, *n* (%)	4 (12.1)	6 (18.2)	0.492
Postoperative ileus, *n* (%)	3 (9.1)	8 (24.2)	0.099
Postoperative hypoxemia, *n* (%)	3 (9.1)	0	0.237
Dizziness	13 (39.4)	10 (30.3)	0.438
Headache	7 (21.2)	7 (21.2)	1.000
Pruritus	0	1 (3.0)	1.000

^a^Continuous variables are presented as means ± SD or median (25th percentile–75th percentile)

^b^Categorical variables are presented as frequency (%)

## Discussion

To the best of our knowledge, this is the first randomized and prospective study comparing the effects of OFA combined with ultrasound-guided ICPB and opioid-based anesthesia in thyroid surgery. These results indicate that this combined strategy is feasible. It may reduce the use of vasoactive drugs during surgery, decrease the incidence of PONV, and improve the quality of postoperative recovery of patients undergoing thyroid surgery. In addition, OFA combined with ultrasound-guided ICPB can significantly reduce the VAS score of patients at rest and during swallowing. Further, it can also reduce the use of rescue analgesics after surgery.

Considering that there is a positive correlation between PONV and the dose of opioids, 0.05–0.2ug/kg/min remifentanil was administered during surgery to control the occurrence of PONV in the control group as much as possible. In addition, postoperative use of dezocine to relieve pain is also an important factor leading to PONV. Despite this, we can still observe that the incidence of PONV is significantly reduced in the OFA group in subgroup analysis. Therefore, we are more inclined to maintain that OFA is a better choice than opioid-based anesthesia in reducing the incidence of PONV. A systematic review and meta-analysis by Franknecht et al. [26] also confirmed this view, and the quality of evidence was high. Due to the long-term antiemetic effects of dexmedetomidine, lidocaine, and s-ketamine, along with the absence of opioids during surgery, the effects of OFA may be verified [6, 27]. PONV is generally considered to be an unfortunate but inherent effect of opioid analgesia. It has been demonstrated that patients are most concerned about avoiding vomiting, ranked even before postoperative pain [25]. The occurrence of PONV has brought pressure on patients and caused the consumption of system resources, including delayed recovery, long-term stay in rehabilitation areas and hospitals, accidental admission, and ultimately increased medical service costs [28]. Therefore, we believe that OFA is a major advantage in the strategy of preventing PONV, especially in high-risk patients [28].

A meta-analysis by Hung et al. [29] concluded that OFA could mitigate postoperative pain after bariatric surgery, which was in line with what we have found. However, Franknecht et al. [26] pointed out that OFA had no effect on pain scores after surgery. In contrast to our results, the pain scores for OFA within 6 h after surgery were lower. This may explain that the analysis included various opioid-free approaches, which indicated that regional block techniques were implemented in not all trials. Thus, we may have obtained lower pain scores within 6 h after surgery as a result of the regional technique of ICPB.

The QoR-40 score was employed to evaluate the influence of OFA on the quality of postoperative recovery. The minimal benefit of value to patients is called the “minimal clinically important difference” [30]. It had been revealed in a study that the minimal clinically important difference for the QoR-40 scores was 6.3 [30]. Our study showed that the OFA resulted in an increase of 7 points in the global QoR-40 score 24 h after surgery. Since the difference between the two groups exceeded the “minimal clinically important difference”, we believed that the improvement of QoR-40 scores in the OFA group was clinically significant. However, a randomized controlled trial conducted on 103 patients undergoing sleeve gastrectomy revealed that there was no significant difference in the QoR-40 score between the opioid-free anesthesia and the opioid-based anesthesia 1 day after surgery [31]. It is unlikely that this analgesic effect will last for more than 24 h after surgery; however, the comfort experience of patients during this early postoperative period may alleviate their pain in the subsequent treatment. In addition, opioids are associated with adverse side effects that negatively affect the postoperative recovery quality of patients. Additionally, the differences in the study population, opioid-free drug dose, and surgical trauma may cause inconsistent results between previous studies and ours. Three patients in the OFA group experienced transient hypoxemia in the PACU. These three patients all suffered from snoring with BMI > 29 kg/m^2^, and higher BMI was an independent risk factor for postoperative hypoxemia. Therefore, we tend to believe that this is due to the patient's own condition rather than the anesthesia method. Nevertheless, there are still several limitations in this study. First, only the parameters 24 h after surgery were compared between both groups, because incision pain and nausea after thyroid surgery mainly affected patients on the first day of recovery [9, 32]. Second, anesthesiologists participating in the trial cannot be blinded to the grouping of the trial due to the major differences in anesthesia methods between the two groups. Lastly, this combination of drugs remains largely unproven. Hence, it is required to monitor intraoperative pain accurately, as well as investigate the benefits of more types of surgery.

## Conclusion

The combination of OFA and ultrasound-guided ICPB is feasible in thyroid surgery. Compared with opioid-based anesthesia, this combined strategy has a lower incidence of PONV, more stable intraoperative hemodynamics condition, more effective postoperative analgesia effect, and better quality of recovery 24 h after surgery. This may be a better alternative technique for anesthesia in thyroid surgery.
